# Transformer Based Binocular Disparity Prediction with Occlusion Predict and Novel Full Connection Layers

**DOI:** 10.3390/s22197577

**Published:** 2022-10-06

**Authors:** Yi Liu, Xintao Xu, Bajian Xiang, Gang Chen, Guoliang Gong, Huaxiang Lu

**Affiliations:** 1 Institute of Semiconductors, Chinese Academy of Sciences, Beijing 100083, China; 2 University of Chinese Academy of Sciences, Beijing 100089, China; 3 School of Microelectronics, University of Science and Technology of China, Hefei 230026, China; 4 Materials and Optoelectronics Research Center, University of Chinese Academy of Sciences, Beijing 200031, China; 5 College of Microelectronics, University of Chinese Academy of Sciences, Beijing 100049, China; 6 Semiconductor Neural Network Intelligent Perception and Computing Technology Beijing Key Laboratory, Beijing 100083, China

**Keywords:** transformer, attention, binocular disparity

## Abstract

The depth estimation algorithm based on the convolutional neural network has many limitations and defects by constructing matching cost volume to calculate the disparity: using a limited disparity range, the authentic disparity beyond the predetermined range can not be acquired; Besides, the matching process lacks constraints on occlusion and matching uniqueness; Also, as a local feature extractor, a convolutional neural network lacks the ability of global context information perception. Aiming at the problems in the matching method of constructing matching cost volume, we propose a disparity prediction algorithm based on Transformer, which specifically comprises the Swin-SPP module for feature extraction based on Swin Transformer, Transformer disparity matching network based on self-attention and cross-attention mechanism, and occlusion prediction sub-network. In addition, we propose a double skip connection fully connected layer to solve the problems of gradient vanishing and explosion during the training process for the Transformer model, thus further enhancing inference accuracy. The proposed model in this paper achieved an EPE (Absolute error) of 0.57 and 0.61, and a 3PE (Percentage error greater than 3 px) of 1.74% and 1.56% on KITTI 2012 and KITTI 2015 datasets, respectively, with an inference time of 0.46 s and parameters as low as only 2.6 M, showing great advantages compared with other algorithms in various evaluation metrics.

## 1. Introduction

The disparity prediction algorithm based on convolutional neural networks builds the matching cost volume by matching the best correspondence between the pixels in the epipolar lines pair. There are still numerous limitations and flaws in the method of calculating disparity: (1) As a local feature extractor, convolutional neural networks lacked the ability to perceive context information. (2) When using a finite disparity range, accurate disparity beyond the specified range cannot be obtained. (3) There were no occlusion or matching uniqueness constraints in the matching process.

With the continuous development of deep learning technologies, the Transformer for sequence-based tasks has shown superior performance over convolutional neural networks in both natural language processing and computer vision. The improvement in algorithm performance is also driving the demand for new terminal applications. As a result, we propose a disparity prediction algorithm based on Transformer to address the problems that exist in the matching method of constructing the matching cost volume and to meet the terminal application requirements of Transformer. Specifically, it includes the Swin-SPP module for feature extraction based on the Swin Transformer, a Transformer disparity matching network based on self-attention and cross-attention mechanisms, and an occlusion prediction sub-network. The disparity prediction algorithm, which is based on the Transformer, solves the matching cost volume problem in the following ways:

First, the disparity range limitation can be removed by utilizing the sequence-to-sequence calculation method of Transformer, avoiding the collision between the camera and objects. Besides, we use the constraint that a pixel in an image should not match multiple pixels in another image as they correspond to the same position in the real scene [1], which has a significant impact on resolving ambiguity and reducing network overfitting. While it is not included in existing methods based on matching cost volume. Last, we use the occlusion prediction model and the adaptive sub-network to predict disparity and the occlusion region, which is more conducive to the downstream tasks such as 3D reconstruction.

Our contributions are outlined as follows: (1) Utilizing the Swin Transformer to extract the features, which enhances the representation ability of the network at extremely microscopic structures. (2) A fully connection layer with double skip connection structure is proposed to solve the problems of gradient disappearance and explosion in Transformer model. (3) Occlusion prediction and adaptive sub-network are used to obtain high-resolution disparity and location of occlusion region.

## 2. Related Work

### 2.1. Stereo Matching

The reference [2] described a range of classic stereo matching algorithms, which match handmade features in multiple pictures and are unable to efficiently cope with high-textured regions, huge uniform featureless regions, and occlusion regions with repeated images. The reference [3] summarized a variety of stereo depth estimation algorithms based on deep learning technology, for example, the reference [4] used four convolutional layer encoders to replace the traditional hand-made feature method, reference [5] compared with reference [4], each layer of encoder added maximum pooling and downsampling, Larger patch and A larger variation in the viewpoint can be handled. A Spatial Pyramid Pooling (SPP) module is added at the end of feature extraction branch. The size of feature grid is fixed by aggregating the features of the last convolutional layer, so the model can handle patches of arbitrary size. The references [4,5] fed the learned features to the top module that calculates the similarity score, The Top Module uses a decision network composed of a fully connected layer and ReLU, which is trained jointly with feature extraction, so that it has the advantage of more accurate accuracy, but the calculation speed is too slow.

The reference [6] used residual network to improve the training of deep network. The model can adaptively adjust the contribution of skipping connections to the network, and achieves better results than the reference [7]. References [8,9,10] expanded the receptive field of the network without loss of resolution and computational efficiency, which solves the problem that increasing the kernel size of CNN to improve the receptive field leads to higher computational load. In addition, the traditional pooling used in the references [4,7] will lead to the loss of accuracy and is not suitable for dense correspondence estimation. Based on this, reference [10] using dilated convolutions, references [8,9] using Spatial Pyramid Pooling (SPP) method to solve the above problems. References [5,11] proposes a network architecture that can deal with multi-scale features, which has the advantage of computing multiple scale features in a single forward propagation. However, the model requires a computational branch for each scale, and the computational resource demand is too high.

A trainable method for calculating the cost has been proposed for regularization and dimensions estimation, in which the designed SGM-Net [12] achieves better performance than the stereo matching cost proposed by Zbontar et al. [7]. However, the streaking artifacts introduced by the SGM method will cause a loss in accuracy. In addition, the method has a high computational memory footprint and cannot process high-resolution images on resource-constrained devices. Further, Schonberger et al. [13] improves the fusion step of SGM and solves the problem of high computational memory usage of SGM-Net.

In recent years, binocular disparity prediction has been applied in many fields. The reference [14] proposed a model that includes the processing of gray-scale images and depth images. They used weighted least squares filtering to preprocess the luminance of grayscale image response and obtained the luminance of grayscale image and disparity information of depth image responses through V1 neuron responses. The reference [15] investigated a real-time Artificial Intelligence road detection system based on binocular vision sensors to improve the trustworthiness of road condition detection. The reference [16] proposed a model for performing continuous stereo matching which introduced a Reusable Architecture Growth (RAG) framework that leverages task-specific neural unit search and architecture growth for continual learning of new scenes.

### 2.2. End-to-End Methods for Stereo Matching

Currently, trainable end-to-end methods to solve the stereo matching problem have been widely studied. Some define depth estimation as a regression problem, using a single encoder-decoder to stack the left and right maps and calculating the disparity map with a regression view [17,18]. Such methods run faster for withdrawing the feature matching module while holding the disadvantage of lacking necessary but hard to access large datasets.

Another end-to-end approach is to imitate the traditional stereo matching method and decompose the problem into multiple stages composed of different modules, such as a multi-branch network with N branches for N input images [17,19,20,21,22,23]. Likewise, PSM-Net [19] uses a Spatial Pyramid Pooling(SPP) module to extract and aggregate multi-dimension features. Knobelreiter et al. [24] propose a combination of CNN and CRF for calculating the matching cost, making better use of training datasets, and achieving better performance. Besides, Xue et al. [25] propose a new RNN-formed CRF method to make the model parameters independent of the number of depth samples. Paschalidou et al. [26] define the inference in MRF as a differentiable function so that back-propagation can be used for end-to-end training.

The method of using multiple 2D convolutions to generate a 3D cost volume is also widely used [17,18,20,21], achieving higher computational efficiency, but this method only captures and aggregates context along the spatial dimension, ignoring the disparity dimension. Additionally, regularized 2D cost maps are calculated by GRU along the depth direction to solve this problem [27].

### 2.3. Supervised and Self-Supervised Method

Dosovitskiy et al. [18] utilize a supervised method to calculate the feature of single-size input in a convolution network and employ the correlation structure and 2D convolutions to calculate cost volume. On the other hand, Yang et al. [28] utilize the self-supervised method and employ Shallow ResNet to compute the input features. Meanwhile, correlation, left features, and segmentation masks are used to calculate the cost volume. The disparity is calculated by regression with encoder-decoder, achieving a significantly smaller error than the supervised method [18]. Additionally, a supervised method is used to calculate features of multiple-size inputs using SPP [29], in which group-wise correlation and stacked hourglass nets are used to calculate cost volume, and soft argmin is employed to compute the disparity, reaching a much more precise than the method [28].

### 2.4. Transformer-Based Structure

All the above work on CNN and other deep learning technologies fails to consider the advantages of Transformer in extracting long sequence information. The Transformer has been used in various fields of artificial intelligence due to its better sequence information processing ability than CNN. The Transformer structure is completely based on the attention mechanism [30]. Unlike the RNN model that accumulates input information continuously, Transformer uses stacking self-attention layers and feedforward layers to replace the recursive structure. With high parallelism, The Transformer is widely used in tasks such as image classification task [31], object recognition [32], image news title generation [33], video understanding [34], and speech recognition task [35]. However, the research results of applying Transformer to depth estimation are rare.

## 3. Architecture

**Figure 1:**
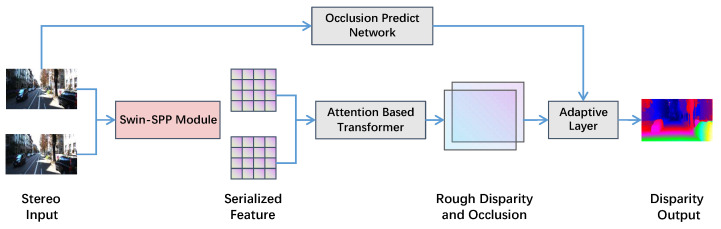
Disparity algorithm framework based on Transformer.

Figure 1 illustrates the overall network architecture of our Transformer-based disparity prediction algorithm. The first part is the Transformer stereo matching backbone network based on self-attention and cross-attention mechanism, which initially utilizes a Swin-SPP feature extraction module with the ability of global information perception to extract features from stereo image pairs and convert them into serialized features. It then uses the Transformer structure to perform self-attention and cross-attention calculations on the extracted feature maps, obtaining the original disparity and the occlusion area. The second part contains occlusion prediction and an adaptive sub-network. The occlusion area label and the left image in the stereo image pair are simultaneously input to the CNN-based occlusion prediction network to obtain the supervised information of the occlusion area, which is further input to the adaptive sub-network with the initial occlusion information obtained by the Transformer. The adaptive sub-network fuses the input image context and semantic information between different epipolar lines, performs disparity optimization, and obtains refined disparity and the position of occluded areas. It distinguishes the disparity information with low confidence of the occluded parts from the reliable ones for non-occluded regions while obtaining disparity information at close range.

The Transformer-based stereo matching algorithm solves the problem of matching cost volume in the following ways: First, the use of the Swin Transformer contributes to expanding the feature extractor’s global information perception ability, which improves the context-sensing ability of the network. Second, the sequence-to-sequence calculation method of the Transformer avoids disparity range limitation, which can make binocular disparity prediction more flexible and avoid collision between camera and object. Third, the match uniqueness constraint is explicitly employed for a pixel in an image should not match multiple pixels in another image [1] because they correspond to the same position in a natural scene. This constraint has a tremendous effect on resolving ambiguity and avoiding overfitting and has not been used in the existing methods based on matching cost volume. The disparity generation based on Transformer adds geometric attribute constraints to binocular disparity prediction in deep learning through optimal transport, including uniqueness constraints. Fourthly, the processing of the occlusion area is included. The occluded region in binocular disparity prediction has no effective disparity, so the algorithm usually inferences the disparity of the occluded region by piecewise smoothness assumption. The occlusion prediction and adaptive sub-network use the occlusion data in the label combined with the context to predict the occlusion region, output the range of the occlusion region, and obtain the confidence estimation and disparity value based on the attention weight, which is more conducive to the disparity prediction scene with high-reliability requirements.

## 4. Stereo Matching Backbone Network Based on Self-Attention and Cross-Attention

In this section, a neural network with an encoder-decoder structure analogous to [36] is designed to extract features from left and right stereo images. The left and right stereoscopic images are concatenated at the channel dimension to construct the input of the encoder network, which facilitates the network to completely use the information of the left and right images in the process of feature coding.

The encoder network comprises a Swin Transformer and a space pyramid pooling module (SPP) [19]. Among them, Swin Transformer, based on a self-attention mechanism, can effectively capture the correlation between different regions of the image, thus better extracting the global features of the input image. Swin Transformer alternately operates self-attention computation and cross-attention computation, which effectively reduces the amount of computation and simultaneously makes the features of different areas interact, eventually enhancing the ability to extract local image information. Since the input image is mapped from a low-dimensional space to high dimension, during which some details are lost, in this chapter, SPP is employed to fuse large-scale-low-dimensional features with small-scale-high-dimensional features, which further enhances the ability of the encoder to charge more details, meanwhile inducing superficial network information interaction pathways to deep network, evading the problem of gradient vanishing to a certain extent during the training process as well as promoting network convergence.

The decoder network, mainly composed of Dense Block [37] and transpose convolution, decrypts the output of SPP from the multi-scale fusing features into a feature map with the exact resolution as the original image. Dense Block primarily realizes the decoding assignment, framed by multiple convolutional hierarchies, in which the input of each convolutional layer is the summation of all previous convolutional layers’ outputs so that the extracted features in each convolutional layer can acquire an essentially lossless transmission. On the other hand, transpose convolution is employed to learn the mapping from high-dimensional features to low-dimensional ones, enhancing the resolution of decoded features and continuously retrieving the detailed features of the original image. At the end of our decoder, another convolutional layer is used to separate the decoded features, eventually acquiring the features of the left and right stereo images.

### 4.1. Skip Connection Structure in Transformer Feedforward Layer

**Figure 2:**
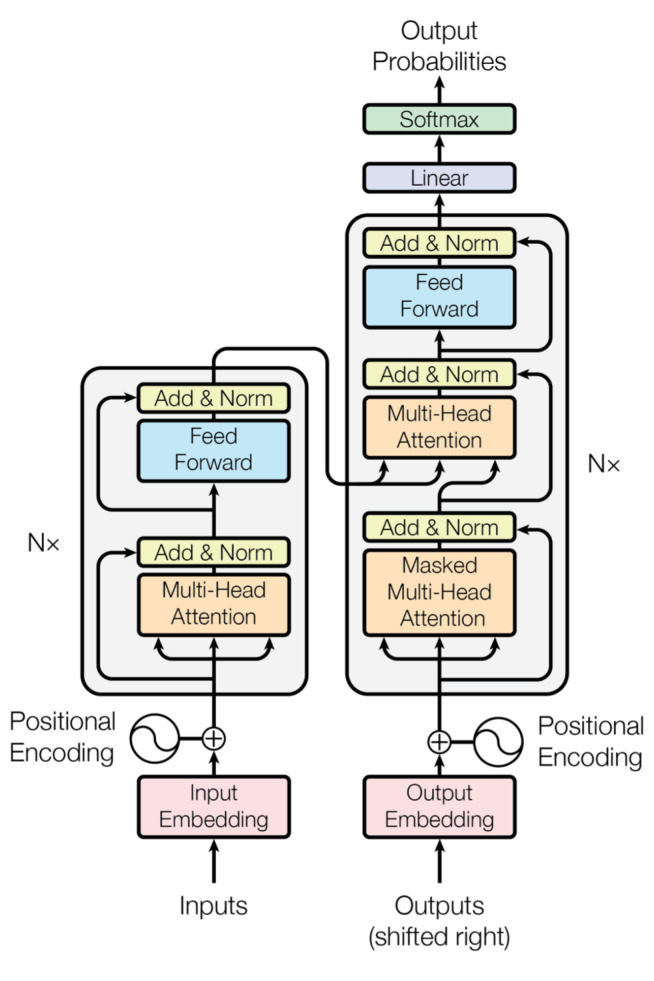
Traditional Transformer structure.

**Figure 3:**
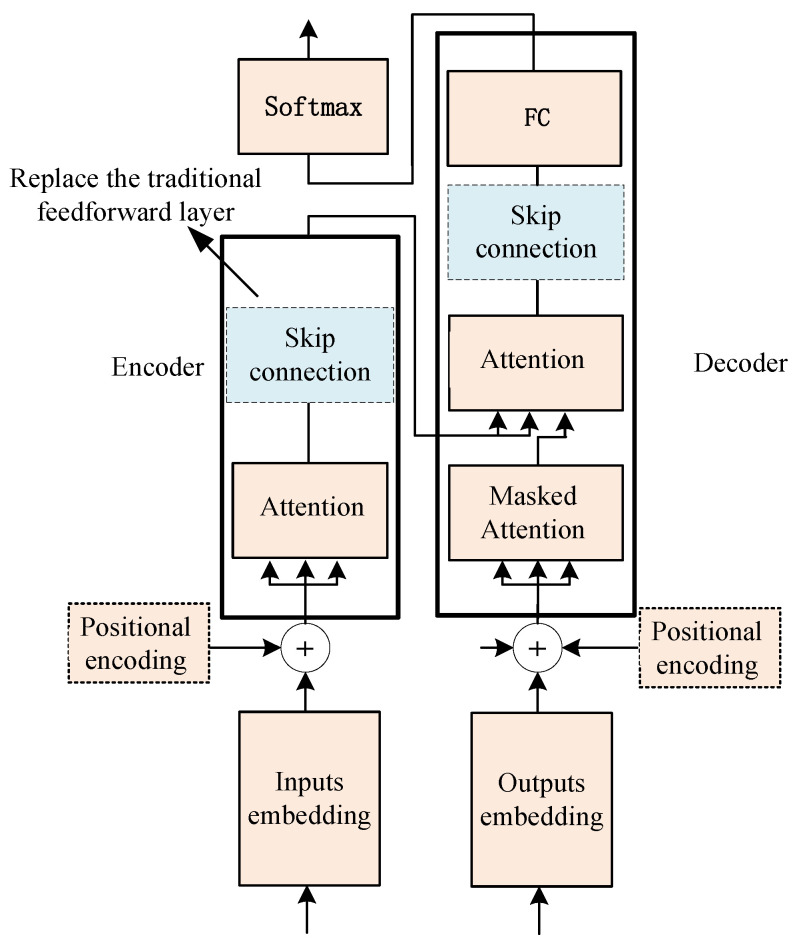
A Transformer structure containing double skip connection.

Figure 2 presents the traditional Transformer structure, in which the feedforward layer introduces nonlinearity while also rendering the issue of training difficulty at the same time. In order to solve this problem, we propose a double skip connection layer fully connected layer to improve the traditional feedforward layer, as shown in Figure 3.

So far, researchers have designed a variety of new skip layer connections, the basic principle of which is to scale the input according to a predetermined value λ so that the scaled input directly adjusts the output, and the linear component of the input is introduced to the output. As shown in (1):

(1) $$y=N\left(\lambda x+F\left(x,W\right)\right)$$

where *y* is the output, *N* is the normalization operation, λ is the scaling factor that connects the input to the output, *x* is the input, *W* is the weight of the neural network, and *F* is the residual block. This formula shows that the output is related to not only the nonlinear feedforward layer but also the input component. Even if the feedforward layer introduces a wrong signal, the output will not drive an error of the complete result due to the input component.

Poole et al. [38] propose a method of using the mean-field theory for solving gradient problems in stochastic networks, who points out that gradient vanishing and exploding problems arise when variance functions of network parameters are in ordered and disordered phases, respectively. Additionally, Schoenholz et al. [39] use mean-field theory to study the maximum depth neural networks can achieve under trainable conditions. Research has also found that operations such as batch normalization and fully connected skip layer structure [40] are conducive to increasing network depth. The influence of layer normalization on Transformer training and inference effect has also been investigated. According to Xiong et al. [40], when the layer normalization is in front of the feedforward layer, the gradient descent is faster, and the inference effect is better than in other positions.

**Figure 4:**
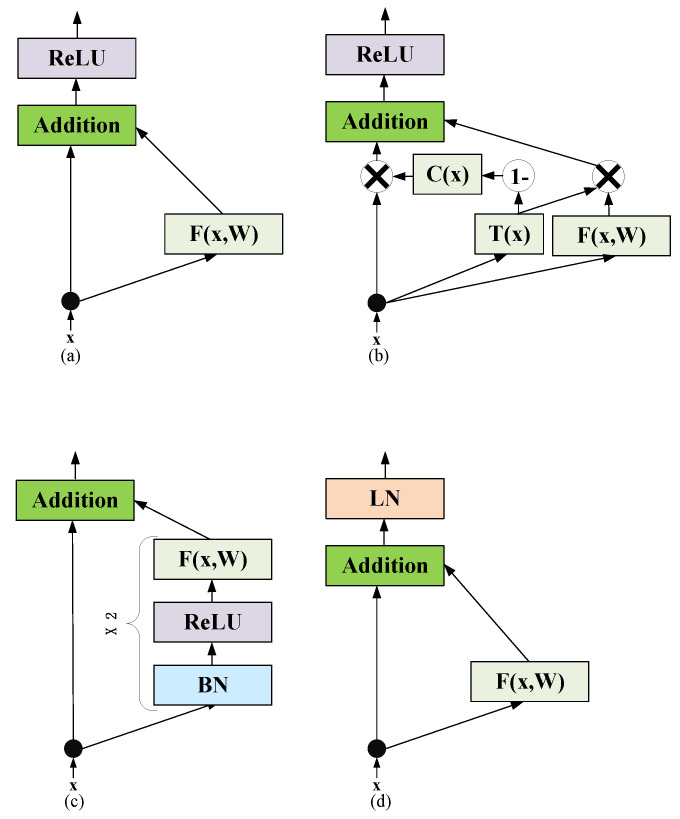
Common skip connection structures.

Figure 4 illustrates several skip layer connection structures, where Figure 4a is a traditional skip layer fully connected structure [41] without scaling the input, Figure 4b is a skip layer structure with a transition gate and a carry gate proposed by [42]. Figure 4c is a structure proposed by [43] in which batch normalization is performed first and the activation function is placed after the full connection layer. Figure 4d is a traditional skip layer fully connected structure with layer normalization for Transformer [30].

The Transformer feedforward layer is a skip layer connection structure, which introduces layer normalization and effectively solves the problem of inadequate gradient propagation in the deep Transformer structure. Layer normalization is the key to achieving satisfactory results in Transformer. Remarkable results have been obtained in several tasks such as machine translation [30], language model [44] after adding layer normalization between residual blocks [45]. Nevertheless, at present, most Transformer training needs warm-ups [46]. Xiong et al. [40] point out that the inference result of the model is strongly correlated with the maximum learning rate and the number of warm-ups; thus, a slight adjustment of the learning rate may cause significant result differences. As a result, it is challenging to encounter the best warm-up parameter of the learning rate, yet it can be alleviated by using the skip connection structure.

**Figure 5:**
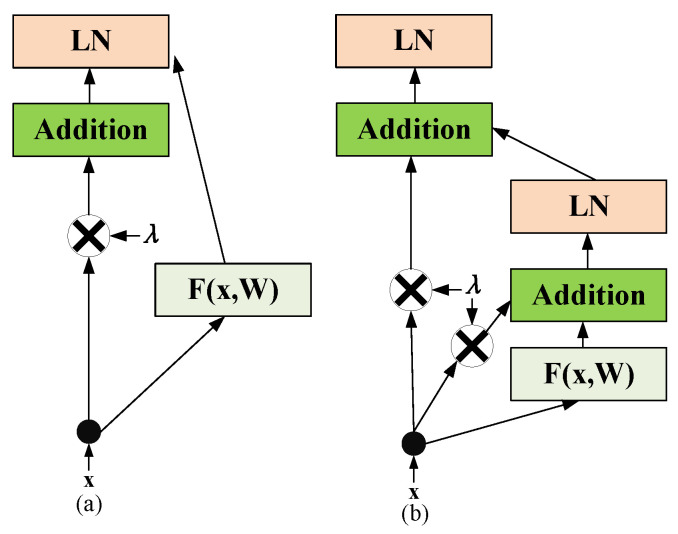
Skip connection structures in Transformer.

Therefore, researchers have designed several new skip layer connection structures for these training problems in Transformer. Figure 5 shows the common skip layer connection structures for Transformer, where Figure 5a is a single-skip fully connected layer structure, and Figure 5b is a recursive skip layer connection with layer normalization proposed by Liu et al. [47]. This structure uses layer normalization to stabilize gradient propagation and employs λ to scale the input. However, the structure in Figure 5 does not take advantage of the layer normalization proposition proposed by Xiong et al. [40], and the scaling parameter λ is not properly set according to the research of Schoenholz et al. [39], and He et al. [43].

**Figure 6:**
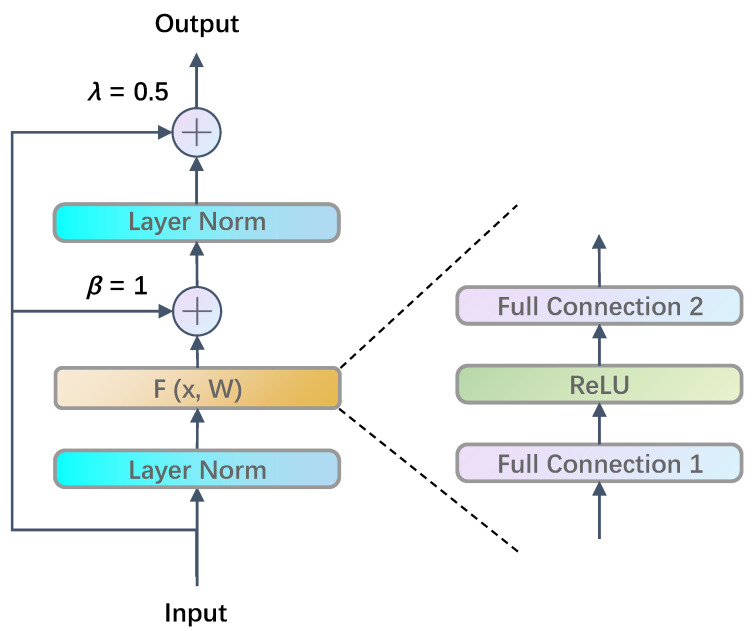
Full connected double skip connection in Transformer.

Existing research results show that the normalization in the feedforward layer should be placed beforehand, the weight λ of input components should not be 1, and the structure of recursive double residual connection benefits. However, current results only consider the advantage of a single variance, and no structure is compatible with multiple advantages. In this paper, a novel double skip connection layer connection structure is designed for Transformer, as shown in Figure 6, which is based on the double-bypass structure, to further improve the quickly risen problem of gradient explosion caused by introducing more input components into the output or setting λ to 2 (more than 1). In our new double skip connection fully connected structure, the layer normalization is set beforehand, and the scaling factors λ and β are set to no more than 1. While introducing more input components to the output, the gradient explosion will not be driven by a too- significant scaling factor, which improves the robustness of the network and ensures stable convergence during training.

### 4.2. Disparity Map Generation Based on Transformer

**Figure 7:**
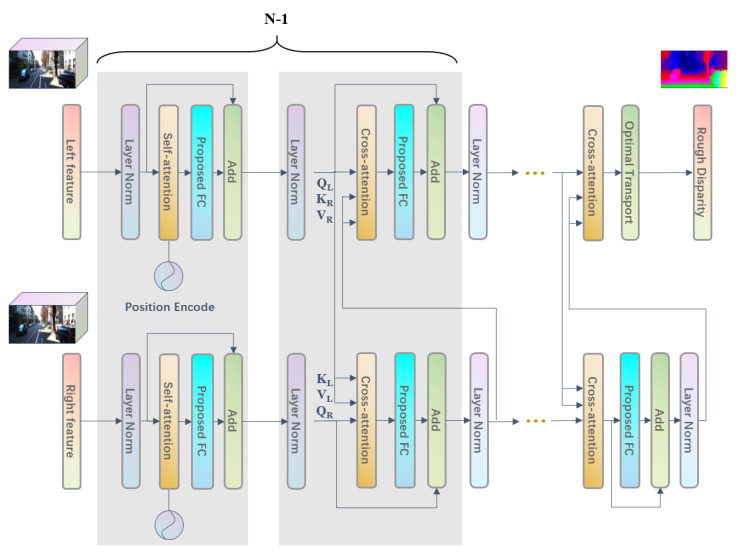
Transformer based on attention.

According to the self-attention and cross-attention mechanism applied to binocular disparity prediction introduced in Section 2.4 and the structure of the Transformer, In this paper, a Transformer structure based on the cross-attention mechanism is designed for disparity map generation, as shown in Figure 7. Unlike the traditional Transformer, the essential module uses two self-attention and two cross-attention modules, as shown in Figure 7 in the curly brackets. In the computation process, the feature map of the left picture is input into the first self-attention module (namely, the encoder). Then, the $K_{L}$ and $V_{L}$ matrices calculated by layer normalization and the $Q_{R}$ matrices obtained by the encoder of the feature map in the right figure are used to compute the cross attention. The same is true for the feature map in the right picture, where the $K_{R}$ and $V_{R}$ matrices and $Q_{L}$ are operated to compute the cross attention, performing a mirror operation, and finally, generating the stereo matching disparity map by stacking this basic module for N-1 times. Since the disparity map of the final output is based on the perspective of the left picture, the fully connected layers and their subsequent layers are canceled in the final output layer of the left picture. After a cross-attention module with a mask and the optimal transmission layer, the final disparity output is obtained.

#### 4.2.1. Attention Mask

In the last cross attention layer, a lower triangular binary mask is introduced to make each pixel in the left figure only processed with the pixel of the same coordinate in the right figure. The spatial position of the stereo camera ensures that after correction, all points satisfy $x_{R}\leq x_{L}$, where $x_{L}\;\mathrm{and}\;x_{R}$ are the projection positions of the same physical point on the left pole line and the right pole line respectively (from left to right is the direction of +x). Thus, the lower triangular binary mask can make the cross-attention layer of the left figure pay attention only to the point *x* of $x\leq x_{L}$ in the right figure.

#### 4.2.2. Explicit Uniqueness Constraints

The above disparity prediction algorithm based on Transformer considers the dense matching of all pixels, but the uniqueness constraint is still missing in the matching process. In order to ensure that each pixel in the right image is assigned to at most one pixel in the left image, Ohta et al. [1] use the uniqueness constraint of stereo matching for the compelled assignment operation, which impedes the gradient propagation during training. To solve this problem, we explicitly modify the weight of cross attention to add the uniqueness constraint to the calculation.

**Figure 8:**
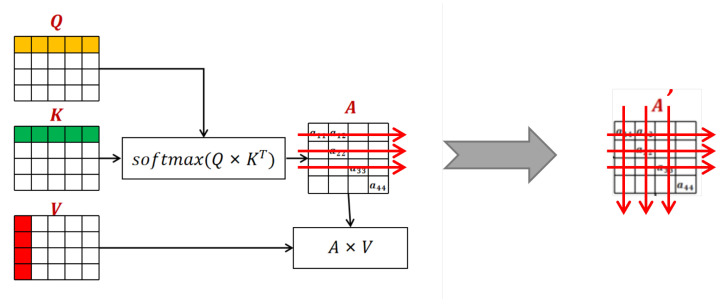
The row and column normalization of attention weights.

As shown in Figure 8, each line of the weight *A* obtained by cross-attention represents the matching probability between each pixel in the left and right image, from which the algorithm locates the optimal matching to calculate the disparity, while *A* in each column of the characterization picture describes the matching probability of each pixel between the right and left image. If normalization is only operated by rows, the constraint relationship between the column will be damaged. Thus, it can not be guaranteed that every pixel on the right will match only one pixel on the left, which violates the matching uniqueness constraints. This constraint can only be satisfied when normalizing the weight matrix *A* with both rows and columns. Therefore, the problem of adding the uniqueness constraint can be transformed into the issue of calculating the normalization matrix by both the column and row, which can further be used to modify the original attention weight matrix.

Since the optimal transmission method has been proven to be differentiable and beneficial for tasks related to sparse features [48] and semantic counterparts [49] matching, the optimal transmission method with differentiable property proposed by Cuturi et al. [50] can be employed to obtain the column and row normalization matrix to impose the uniqueness constraint instead of the forced allocation strategy. The optimal transmission method acquires the optimal coupling matrix $A^{\prime }$ by solving the following equation:

(2) $$\begin{array}{cc} \; & A^{\prime }=\underset{A^{\prime }\in R_{+}^{I_{w}\times I_{w}}}{\mathrm{argmin}}\sum_{i,j=1}^{I_{w},I_{w}}A^{\prime }ijCij \end{array}$$

(3) $$\begin{array}{cc} \; & \;s.t.\;A^{\prime }1_{I_{w}}=a,A^{\prime T}1_{I_{w}}=b \end{array}$$

where *a* and *b* are two marginal distributions with the given length of $I_{w}$, *C* is the matching cost matrix with a size of $I_{w}\times I_{w}$. The values in $A^{\prime }$ represent the probability of matching. If two marginal distributions a,b are identical, then $A^{\prime }$ is the optimal allocation problem, which introduces a soft uniqueness constraint [51] and decreases the uncertainty [49].

#### 4.2.3. Preliminary Disparity Map Generation

When generating preliminary disparity maps, disparity matching based on Transformer cannot directly use a method based on the weighted summation of all candidate disparity values, so an improved winner-takes-all method [52] is used for disparity regression. Foremost, locate the most likely match (denoted as *K*) from the optimal transmission allocation matrix $A^{\prime }$. Next, create a 3×3 pixel computation window W(k) around the match and renormalize the matching probability in the window of 3×3 pixels to obtain the original disparity. The candidate disparity d(k) can be obtained by the weighted summation operation of the original disparity as follows.

(4) $$\begin{array}{c} \tilde{p}i=\frac{pi}{\sum_{i\in W(k)}p_{i}} \end{array}$$

(5) $$\begin{array}{c} d\left(k\right)=\sum_{i\in W(k)}d_{i}{\tilde{p}}_{i} \end{array}$$

where I∈W(k); *P* represents the matching probability in the allocation matrix $A^{\prime }$, and the sum of the probabilities in this 3×3 window represents the estimate of the confidence under the current match in the network.

## 5. Occlusion Prediction and Adaptive Sub-Networks

**Figure 9:**
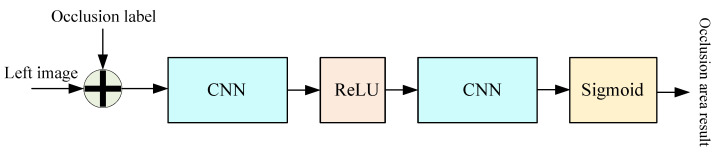
Occlusion area calculation.

In order to alleviate the lack of contextual correlation information on the original disparity and occlusion map among multiple polar lines caused by regression only on polar lines, this section takes the image with cross-polar line information as input condition, using convolution calculation to accommodate the estimated value. Figure 9 exhibits the sub-network used to improve the calculation effect of the occlusion region, composed of the convolution module, ReLU activation function, and Sigmoid activation function module. The original disparity and occlusion map are first connected with the left figure on the channel dimension during the computation procedure. Then, two convolution blocks and the ReLU activation function layer are used to aggregate the occlusion information. Eventually, the location estimation of the occlusion region is computed by a Sigmoid activation function. After determining the location of the occlusion, to solve the problem of pixel unmatching, based on our occlusion prediction sub-network, the matching cost matrix $A^{\prime }$ is extended by the learnable parameters representing the cost of mismatched pixels proposed by Sarlin et al. [48].

**Figure 10:**
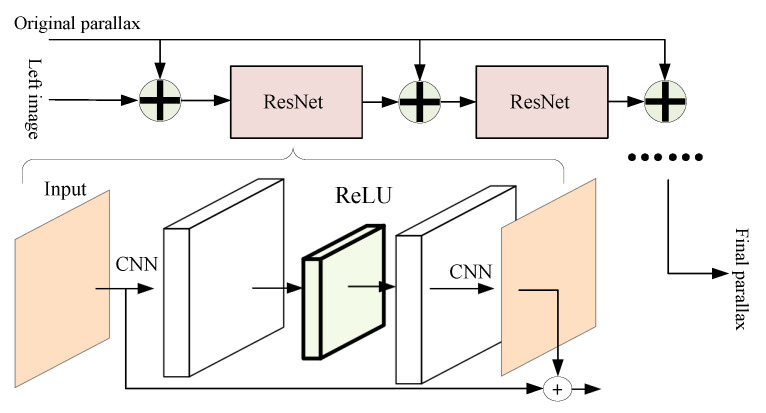
Adaptive sub-network.

Figure 10 shows an adaptive sub-network with a long-skip layer connection for optimizing disparity results. To obtain better information flow [53], the channel size of the residual module in our network is expanded by convolution before the ReLU activation function layer and recovered after calculation. The original disparity and the calculated disparity are aggregated. Besides, to increase the depth of the network and enhance the processing ability, this structure is stacked several times, and the results are concatenated with the initial disparity through the long-skip connection structure, obtaining satisfactory disparity prediction results.

## 6. Experiments

In this section, we conduct experiments on the binocular disparity prediction method based on Transformer to prove the effectiveness of the algorithm and analyze its pros and cons. The algorithm is mainly implemented based on the deep learning framework PyTorch, and the software and hardware experimental platforms used in the experiment are shown in Table 1.

For the network structure, we use 6 groups of self-attention and cross-attention layers, the channel dimension $C_{e}$ of the feature descriptor $e_{I}$ is set to 128, and the number of heads is set to 4 to ensure the light weight of the network. For training hyperparameter Settings, we use Adam with a weight decay of 1e−4 as the optimizer. The feature extractor and Transformer were pre-trained on the DispNet/FlowNet2.0 dataset with a fixed learning rate of 1e−4 for 15 epochs with a batch size of 1. Occlusion prediction and adaptive sub-networks are pre-trained using a learning rate of 2e−4. For benchmarking the KITTI dataset, we fine-tune the pre-trained model for 400 epochs using an optimizer with an exponential learning rate, with a weight decay of 0.99. For data augmentation, in addition to figure-consistent random cropping, vertical movement, and horizontal rotation, to make images from the data set better simulate authentic stereo images, we also employ the asymmetry augmentation operations to the left and right images, including RGB transformation, brightness transformation, contrast transformation and adding Gaussian noise.

### 6.1. Evaluation Methord

For the quantitative evaluation of the disparity map, EPE (Absolute Error) and 3PE (Percentage Error Greater than 3px) are used as evaluation indexes. Additionally, because the occlusion region can be predicted, IoU(Intersection over Union) index is used to evaluate the network’s performance in predicting the occlusion region.

**Figure 11:**
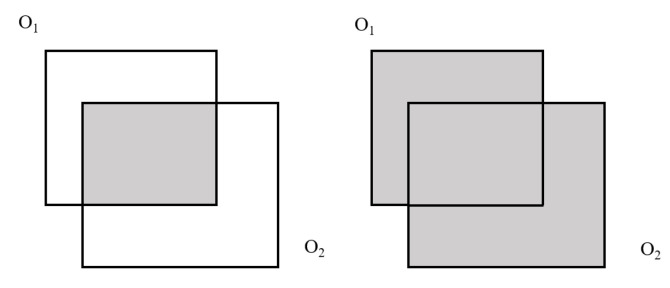
Intersection and union of predicted bounding box and the real one.

IoU is often used in object detection tasks to evaluate the proportion of coincidence between the border predicted by the network and the authentic border, as shown in Figure 11. IoU is calculated as follows:

(6) $$\begin{array}{c} IoU=\frac{O_{1}\cap O_{2}}{O_{1}\cup O_{2}} \end{array}$$

where $O_{1}$ and $O_{2}$ are the predicted border and the natural border of the network, respectively. For the prediction of the occlusion region, $O_{1}$ describes the occlusion region predicted by the network, and $O_{2}$ defines the natural occlusion region.

### 6.2. DispNet/FlowNet2.0 Dataset

Since the occlusion prediction sub-network is included, a dataset containing occlusion data with ground truth labels is required for training. Though SceneFlow [17] dataset provides a large number of synthetic datasets containing random objects, it does not provide occlusion information with the label. This section uses the DispNet/FlowNet2.0 dataset rendered by a 3D engine. The training dataset contains 21,818 images with a resolution of 960 × 540, and the test dataset contains 4248 images of the same size. The maximum disparity of each dataset is 602 pixels and 469 pixels, respectively.

**Figure 12:**
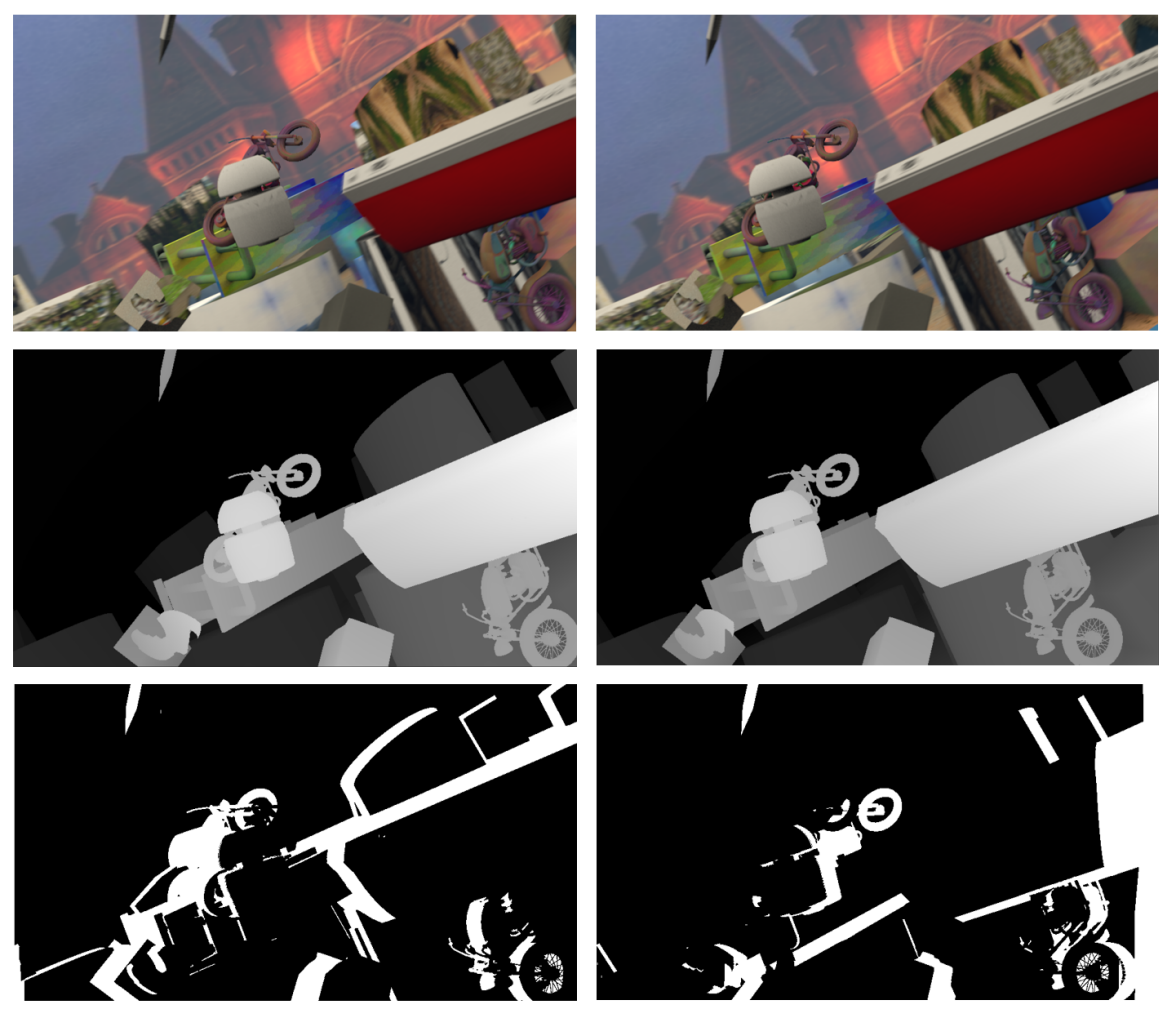
Elements in the DispNet/FlowNet2.0 dataset.

The DispNet/FlowNet2.0 dataset contains the stereo image pairs of the left and right images, the ground truth value of the disparity, and the blocking label. These six images constitute an element in the DispNet/FlowNet2.0 dataset, as shown in Figure 12. The occlusion label data comprises black (0) and white (255). The white areas represent the occlusion caused by the difference between the left and right view angles in the corresponding position of the image, through which the disparity information cannot directly be acquired by matching.

**Figure 13:**
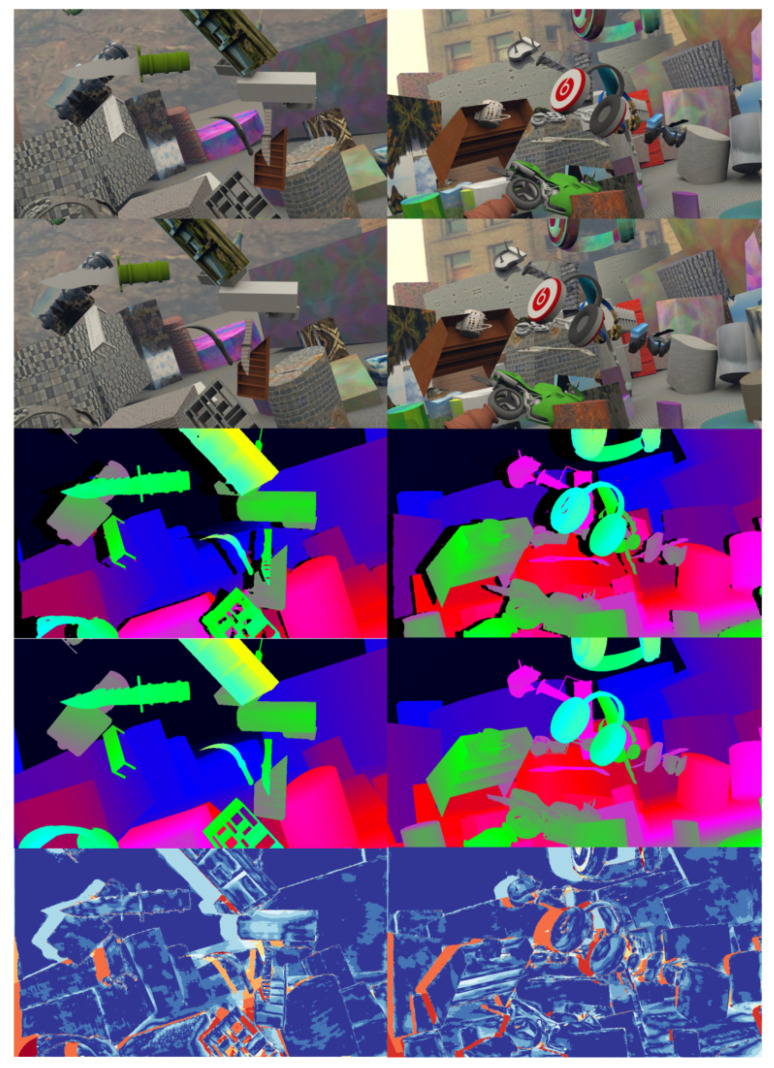
Splitting the default training and testing sets of DispNet/FlowNet2.0 for pre-training.

For pre-training, we split the default training and test sets of DispNet/FlowNet2.0. Parts of the experimental results are shown in Figure 13, in which there are two different columns of data, where the first and second rows represent the input left and right stereo image pairs, and the third row illustrates the network prediction results, the fourth row denotes the real disparity, and the last row is the thermal error map.

**Figure 14:**
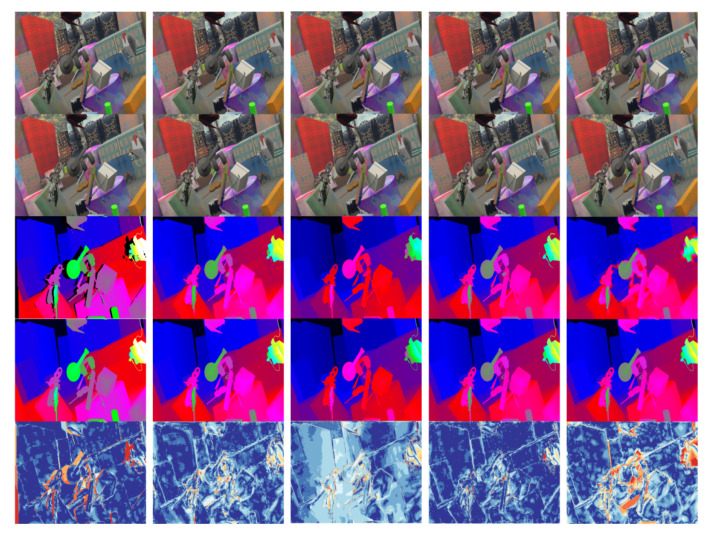
The comparison between out algorithm and other deep learning-based algorithms.

Figure 14 shows the comparison between the algorithm in this section and other deep learning-based algorithms. For fair, the comparison of the occlusion area is removed, so the color of the occlusion area displayed in the thermal error map is warm. The results of Figure 14 are analyzed as follows:

1. Except for the occlusion area, the algorithm in this chapter has a cooler overall tone, indicating a higher accuracy.
2. Our algorithm has a sharper and clearer boundary in the edge area.
3. Our algorithm has a strong representation ability at microscopic structures, which is not exhibited in other methods.
4. Our algorithm presents uniform changes in non-textured areas with disparity gradient (such as tilted surfaces) and has less false matching.

Table 2 shows the quantitative analysis results in the DispNet/FlowNet2.0 dataset and the quantitative analysis results of other binocular disparity prediction algorithms under the same evaluation criteria. The missing data is partly because the method does not analyze the corresponding index, or the algorithm results do not include this index. The average value obtained from 10 repeated experiments was used to evaluate the index in testing the algorithm. The examination of the running time is based on the simulation experiment platform in this section. However, this index is directly related to the CPU performance; Thus, it is only used to compare our algorithms. For other running platforms, it is only for reference.

Table 2 exhibits that the method in this chapter is better than other methods in terms of endpoint error index EPE and 3-pixel error 3PE. Compared with PSMNet, GC-Net, and other high precision disparity computing networks, our algorithm’s number of parameters and running time have significant advantages. While on current platforms, the running time is not a tremendous advantage over lightweight algorithms, running in real-time with the acceleration of the new Transformer terminals in the future is possible.

### 6.3. KITTI Benchmark

**Figure 15:**
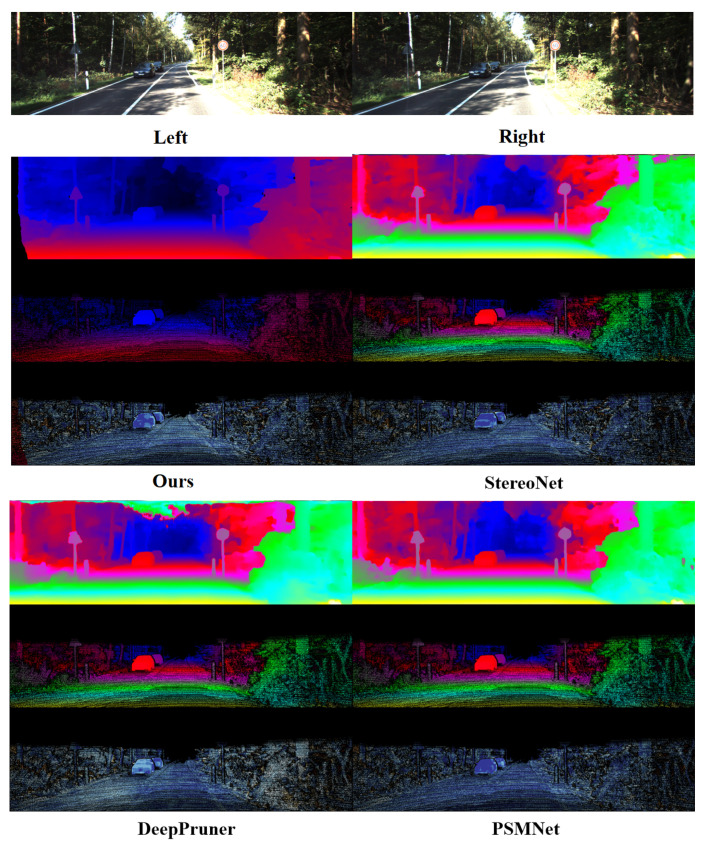
The left and right stereo images of the natural scene in the KITTI dataset.

**Figure 16:**
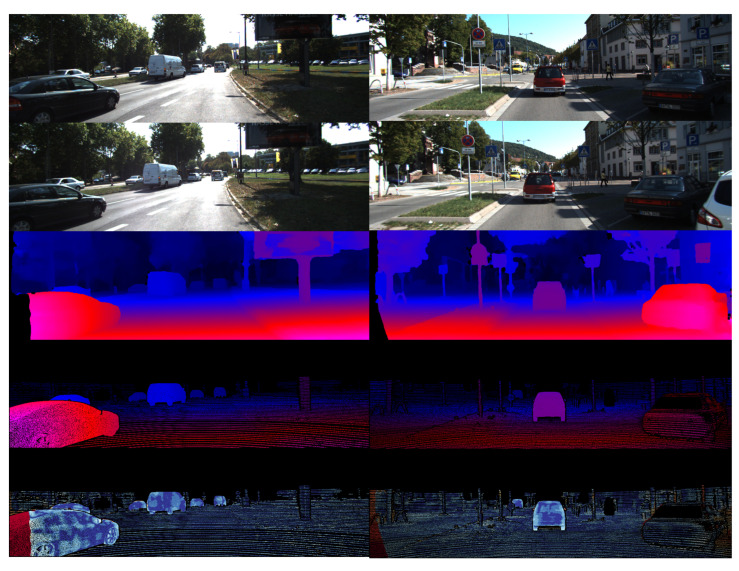
Partial experimental results on KITTI dataset based on our algorithm.

The DispNet/FlowNet2.0 dataset used in the previous section is an extensive collection of data containing disparity information generated by a 3D virtual engine, from which the disparity prediction algorithm based on deep learning can acquire the preliminary disparity prediction ability. Since it is difficult to obtain the dense disparity information, our experiment adopts the transfer learning method in the dataset of natural scenes. The KITTI benchmark consists of two datasets, KITTI 2015 [54] and KITTI 2012, which are objective street view datasets collected by an on-board camera, each containing 200 images with a resolution of 1242 × 375 and a maximum disparity of 192. The left and right stereo images of the natural scene in the dataset are shown in Figure 15. For the evaluation of the KITTI benchmark, KITTI 2012 and 2015 datasets are employed for training, and 20 images are randomly chosen for visual verification. Partial experimental results are shown in Figure 16, which exhibits two different data columns with the same arrangement as the experimental results on the SceneFlow dataset in the previous section.

Figure 15 compares the algorithm in this section with other deep-learning-based algorithms. The comparative experiment results in Figure 15 are analyzed as follows:

1. Our algorithm integrates the occlusion processing module, so the parts with higher thermal error values are less in the challenging area, indicating higher overall accuracy.
2. Compared with other algorithms, our algorithm has a sharper and clearer boundary for the regular region with a small area and obvious boundary (triangle and circular signs in the Figure) while to a prominent extent maintaining the shape characteristics of the regular region with a small area.
3. The algorithm in this chapter has better disparity fitting in the remote area. There is no obvious prediction error for scenes like the sky, and it has less mismatching than other algorithms.

Table 3 and Table 4 exhibit the quantitative results of the algorithm in this section and other binocular disparity prediction algorithms on KITTI under the same evaluation criteria. The average value obtained from 10 repeated experiments was employed to evaluate the testing index. In this section, the KITTI 2012 and KITTI 2015 datasets are mixed, and then the trainset and testset are randomly selected from the mixed dataset to conduct experiments as the final experimental results of the algorithm. The experimental results are shown in the last line of Table 4. Because the KITTI benchmark evaluates the performance of algorithms in natural scenes, the absolute error EPE has little application value in practice; Thus, many other algorithms do not evaluate the EPE index.

By analyzing the data in Table 3 and Table 4, it is evident that the method presented in this chapter is still better than other methods in EPE. Although the 3-pixel error in KITTI 2012 dataset is not significantly reduced, it is significantly reduced in KITTI 2015 dataset.

The previous qualitative analysis indicates that this chapter’s modification of disparity prediction by the algorithm mainly focuses on the areas with fine structure. Since such areas account for a relatively small proportion and current advanced algorithms can produce high-quality disparity, the contribution of this part to the overall accuracy is not particularly obvious.

### 6.4. Generalization Ability

To verify the generalization performance of the algorithm on other datasets and to confirm the non-contingency of the results, the binocular disparity prediction algorithm based on Transformer is directly evaluated on the new target dataset after pre-training on the DispNet/FlowNet2.0 dataset. The weights are not fine-tuned by transfer learning on the corresponding datasets. The quantitative analysis of experimental results is shown in Table 5, which enumerates the network models with confident generalization performance while the ones with unsatisfactory results are not listed. As seen from the Table, although the accuracy is moderately reduced after transfer learning, the algorithm in this chapter has a strong generalization ability, and the disparity output is not a simple fitting for the dataset. Hence, our algorithm shows significant advantages compared with other algorithms in various evaluation indexes.

## 7. Conclusions

Aiming at the demand of disparity prediction with close range and high reliability, we propose a binocular disparity prediction algorithm based on Transformer for terminal AI chips that efficiently support Transformer. For disparity matching, a feature extraction module Swin-SPP based on Swin Transformer is proposed, which enables feature extraction to acquire global information sensing ability. A disparity matching backbone network based on self-attention and cross-attention mechanism is designed to solve the problem of missing matching uniqueness constraints and improve the range of disparity capture. Additionally, the occlusion prediction and adaptive sub-network are employed to predict the occlusion region combined with the context, and the high-quality disparity output is obtained. Our design uses the double skip connection fully connected structure, which shows a positive gain effect on the robustness of the network and a stable convergence in training, and therefore solves the problems of gradient vanishing and explosion in the training of the Transformer model.

Our algorithm with a parameter of 2.6 M and a running time of 0.46 s, obtained an EPE of 0.47 and a 3PE of 1.41% on the DispNet/FlowNet2.0 dataset, obviously better than other methods. Besides, on KITTI 2012 and KITTI 2015 datasets, our model achieved an EPE of 0.57 and 0.61, and a 3PE of 1.74% and 1.56%, respectively, showing great advantages compared with other algorithms. The experimental results demonstrate that the algorithm can be calculated within the theoretical maximum disparity range and has a relatively top performance on Scene-Flow and KITTI datasets. Compared with other algorithms, our algorithm has a sharper and clearer boundary in the edge area and has a strong representation ability in microscopic structures. Moreover, the generalization performance on other datasets without transfer learning is satisfactory, which provides algorithm support for the open terminal AI chip that efficiently supports Transformer and has a good application prospect. In the future work, we will lightweight the binocular disparity prediction algorithm based on Transformer to reduce the difficulty of algorithm deployment.

## Figures and Tables

**Table 1:** The software and hardware platform required for Transformer algorithm.

Platform	Parameters
CPU	Intel Core i7-10700 @2.9 GHz
GPU	NVIDIA GTX 2080Ti
Memory	DDR4 3200 Hz 16 G
Operating System	Ubuntu 18.04 LTS
Deep Learning Framework	PyTorch 1.7.1

**Table 2:** Quantitative evaluation on the DispNet/FlowNet2.0 dataset.

	EPE	3PE%	Occulution IOU%	Parameters	Runtime
PSMNet	1.25	3.31	—	5.2 M	0.59 s
AnyNet	3.19	—	—	40,000	97.3 ms
DeepPruner	0.86	2.13	—	N/A	182 ms
AANet	0.87	—	—	N/A	62 ms
GC-Net	2.51	9.34	—	3.5 M	0.95 s
Ours	0.47	1.41	98.04	2.6 M	0.46 s

**Table 3:** Quantitative evaluation on the KITTI 2012 dataset.

	EPE	3PE/%	Occlusion IOU/%	Parameters	Runtime
PSMNet	0.6	1.89	—	5.2 M	0.50 s
AnyNet	—	6.10	—	40,000	97.3 ms
DeepPruner	—	2.03	—	N/A	180 ms
AcfNet	0.58	1.78	—	5.6 M	0.48 s
GC-Net	0.70	2.30	—	3.5 M	0.9 s
Ours	0.57	1.74	98.80	2.6 M	0.46s

**Table 4:** Quantitative evaluation on the KITTI 2015 dataset.

	EPE	3PE/%	Occlusion IOU/%	Parameters	Runtime
PSMNet	—	2.33	—	5.2 M	0.50 s
AnyNet	—	6.20	—	40,000	97.3 ms
DeepPruner	—	2.15	—	N/A	180 ms
AcfNet	—	1.89	—	5.6 M	0.48 s
GC-Net	—	2.87	—	3.5 M	0.9 s
Ours	0.6869	2.04	99.86	2.6 M	0.46 s
Ours*	0.6098	1.56	99.87	2.6 M	0.46 s

**Table 5:** Generalization performance test.

	Middlebury			KITTI		
	EPE	3pix Error	Occlusion IOU	EPE	3pix Error	Occlusion IOU
PSMNet	3.05	12.96	—	6.56	27.79	—
GwcNet	1.89	8.59	—	2.21	12.60	—
AANet	2.19	12.80	—	1.99	12.42	—
Ours	2.23	6.09	95.5%	1.40	5.74	98.7%

## Data Availability

The datasets we used in this study are the KITTI and SceneFlow datasets, and they are openly available in http://www.cvlibs.net/datasets/kitti/ (accessed on 7 August 2022). and https://lmb.informatik.uni-freiburg.de/resources/datasets/SceneFlowDatasets.en.html (accessed on 7 August 2022).

## References

[1] Ohta Y., Kanade T. (1985). Stereo by intra-and inter-scanline search using dynamic programming. IEEE Trans. Pattern Anal. Mach. Intell..

[2] Scharstein D., Szeliski R. (2002). A taxonomy and evaluation of dense two-frame stereo correspondence algorithms. Int. J. Comput. Vis..

[3] Laga H., Jospin L.V., Boussaid F., Bennamoun M. (2020). A survey on deep learning techniques for stereo-based depth estimation. IEEE Trans. Pattern Anal. Mach. Intell..

[4] Zbontar J., LeCun Y. (2016). Stereo matching by training a convolutional neural network to compare image patches. J. Mach. Learn. Res..

[5] Zagoruyko S., Komodakis N. Learning to compare image patches via convolutional neural networks. Proceedings of the IEEE Conference on Computer Vision and Pattern Recognition.

[6] Shaked A., Wolf L. Improved stereo matching with constant highway networks and reflective confidence learning. Proceedings of the IEEE Conference on Computer Vision and Pattern Recognition.

[7] Zbontar J., LeCun Y. Computing the stereo matching cost with a convolutional neural network. Proceedings of the IEEE Conference on Computer Vision and Pattern Recognition.

[8] Park H., Lee K.M. (2016). Look wider to match image patches with convolutional neural networks. IEEE Signal Process. Lett..

[9] Ye X., Li J., Wang H., Huang H., Zhang X. (2017). Efficient stereo matching leveraging deep local and context information. IEEE Access.

[10] Fu H., Gong M., Wang C., Batmanghelich K., Tao D. Deep ordinal regression network for monocular depth estimation. Proceedings of the IEEE Conference on Computer Vision and Pattern Recognition.

[11] Chen Z., Sun X., Wang L., Yu Y., Huang C. A deep visual correspondence embedding model for stereo matching costs. Proceedings of the IEEE International Conference on Computer Vision.

[12] Seki A., Pollefeys M. Sgm-nets: Semi-global matching with neural networks. Proceedings of the IEEE Conference on Computer Vision and Pattern Recognition.

[13] Schonberger J.L., Sinha S.N., Pollefeys M. Learning to fuse proposals from multiple scanline optimizations in semi-global matching. Proceedings of the European Conference on Computer Vision (ECCV).

[14] Zhang Q., Lin C., Li F. (2021). Application of binocular disparity and receptive field dynamics: A biologically-inspired model for contour detection. Pattern Recognit..

[15] Xie Q., Hu X., Ren L., Qi L., Sun Z. (2022). A Binocular Vision Application in IoT: Realtime Trustworthy Road Condition Detection System in Passable Area. IEEE Trans. Ind. Inform..

[16] Zhang C., Tian K., Fan B., Meng G., Zhang Z., Pan C. Continual Stereo Matching of Continuous Driving Scenes With Growing Architecture. Proceedings of the IEEE/CVF Conference on Computer Vision and Pattern Recognition.

[17] Mayer N., Ilg E., Hausser P., Fischer P., Cremers D., Dosovitskiy A., Brox T. A large dataset to train convolutional networks for disparity, optical flow, and scene flow estimation. Proceedings of the IEEE Conference on Computer Vision and Pattern Recognition.

[18] Dosovitskiy A., Fischer P., Ilg E., Hausser P., Hazirbas C., Golkov V., Van Der Smagt P., Cremers D., Brox T. Flownet: Learning optical flow with convolutional networks. Proceedings of the IEEE International Conference on Computer Vision.

[19] Chang J.R., Chen Y.S. Pyramid stereo matching network. Proceedings of the IEEE Conference on Computer Vision and Pattern Recognition.

[20] Pang J., Sun W., Ren J.S., Yang C., Yan Q. Cascade residual learning: A two-stage convolutional neural network for stereo matching. Proceedings of the IEEE International Conference on Computer Vision Workshops.

[21] Liang Z., Feng Y., Guo Y., Liu H., Chen W., Qiao L., Zhou L., Zhang J. Learning for disparity estimation through feature constancy. Proceedings of the IEEE Conference on Computer Vision and Pattern Recognition.

[22] Kendall A., Martirosyan H., Dasgupta S., Henry P., Kennedy R., Bachrach A., Bry A. End-to-end learning of geometry and context for deep stereo regression. Proceedings of the IEEE International Conference on Computer Vision.

[23] Nie G.Y., Cheng M.M., Liu Y., Liang Z., Fan D.P., Liu Y., Wang Y. Multi-level context ultra-aggregation for stereo matching. Proceedings of the IEEE/CVF Conference on Computer Vision and Pattern Recognition.

[24] Knobelreiter P., Reinbacher C., Shekhovtsov A., Pock T. End-to-end training of hybrid CNN-CRF models for stereo. Proceedings of the IEEE Conference on Computer Vision and Pattern Recognition.

[25] Xue Y., Chen J., Wan W., Huang Y., Yu C., Li T., Bao J. Mvscrf: Learning multi-view stereo with conditional random fields. Proceedings of the IEEE/CVF International Conference on Computer Vision.

[26] Paschalidou D., Ulusoy O., Schmitt C., Van Gool L., Geiger A. Raynet: Learning volumetric 3d reconstruction with ray potentials. Proceedings of the IEEE Conference on Computer Vision and Pattern Recognition.

[27] Yao Y., Luo Z., Li S., Shen T., Fang T., Quan L. Recurrent mvsnet for high-resolution multi-view stereo depth inference. Proceedings of the IEEE/CVF Conference on Computer Vision and Pattern Recognition.

[28] Yang G., Zhao H., Shi J., Deng Z., Jia J. Segstereo: Exploiting semantic information for disparity estimation. Proceedings of the European Conference on Computer Vision (ECCV).

[29] Guo X., Yang K., Yang W., Wang X., Li H. Group-wise correlation stereo network. Proceedings of the IEEE/CVF Conference on Computer Vision and Pattern Recognition.

[30] Vaswani A., Shazeer N., Parmar N., Uszkoreit J., Jones L., Gomez A.N., Kaiser Ł., Polosukhin I. (2017). Attention is all you need. Adv. Neural Inf. Process. Syst..

[31] Lanchantin J., Wang T., Ordonez V., Qi Y. General multi-label image classification with transformers. Proceedings of the IEEE/CVF Conference on Computer Vision and Pattern Recognition.

[32] Carion N., Massa F., Synnaeve G., Usunier N., Kirillov A., Zagoruyko S. End-to-end object detection with transformers. Proceedings of the European Conference on Computer Vision.

[33] Tran A., Mathews A., Xie L. Transform and tell: Entity-aware news image captioning. Proceedings of the IEEE/CVF Conference on Computer Vision and Pattern Recognition.

[34] Bertasius G., Wang H., Torresani L. (2021). Is space-time attention all you need for video understanding. arXiv.

[35] Chen X., Wu Y., Wang Z., Liu S., Li J. Developing real-time streaming transformer transducer for speech recognition on large-scale dataset. Proceedings of the ICASSP 2021–2021 IEEE International Conference on Acoustics, Speech and Signal Processing (ICASSP).

[36] Liu X., Zheng Y., Killeen B., Ishii M., Hager G.D., Taylor R.H., Unberath M. Extremely dense point correspondences using a learned feature descriptor. Proceedings of the IEEE/CVF Conference on Computer Vision and Pattern Recognition.

[37] Huang G., Liu Z., Van Der Maaten L., Weinberger K.Q. Densely connected convolutional networks. Proceedings of the IEEE Conference on Computer Vision and Pattern Recognition.

[38] Poole B., Lahiri S., Raghu M., Sohl-Dickstein J., Ganguli S. (2016). Exponential expressivity in deep neural networks through transient chaos. Adv. Neural Inf. Process. Syst..

[39] Schoenholz S.S., Gilmer J., Ganguli S., Sohl-Dickstein J. (2017). Deep information propagation. arXiv.

[40] Xiong R., Yang Y., He D., Zheng K., Zheng S., Xing C., Zhang H., Lan Y., Wang L., Liu T. On layer normalization in the transformer architecture. Proceedings of the International Conference on Machine Learning.

[41] He K., Zhang X., Ren S., Sun J. Deep residual learning for image recognition. Proceedings of the IEEE Conference on Computer Vision and Pattern Recognition.

[42] Srivastava R.K., Greff K., Schmidhuber J. (2015). Highway networks. arXiv.

[43] He K., Zhang X., Ren S., Sun J. Identity mappings in deep residual networks. Proceedings of the European Conference on Computer Vision.

[44] Dai Z., Yang Z., Yang Y., Carbonell J., Le Q.V., Salakhutdinov R. (2019). Transformer-xl: Attentive language models beyond a fixed-length context. arXiv.

[45] Wang Q., Li B., Xiao T., Zhu J., Li C., Wong D.F., Chao L.S. (2019). Learning deep transformer models for machine translation. arXiv.

[46] Liu L., Jiang H., He P., Chen W., Liu X., Gao J., Han J. (2019). On the variance of the adaptive learning rate and beyond. arXiv.

[47] Liu F., Ren X., Zhang Z., Sun X., Zou Y. (2021). Rethinking Skip Connection with Layer Normalization in Transformers and ResNets. arXiv.

[48] Sarlin P.E., DeTone D., Malisiewicz T., Rabinovich A. Superglue: Learning feature matching with graph neural networks. Proceedings of the IEEE/CVF Conference on Computer Vision and Pattern Recognition.

[49] Liu Y., Zhu L., Yamada M., Yang Y. Semantic correspondence as an optimal transport problem. Proceedings of the IEEE/CVF Conference on Computer Vision and Pattern Recognition.

[50] Cuturi M. (2013). Lightspeed computation of optimal transportation distances. Adv. Neural Inf. Process. Syst..

[51] Peyré G., Cuturi M. (2019). Computational optimal transport: With applications to data science. Found. Trends Mach. Learn..

[52] Tulyakov S., Ivanov A., Fleuret F. (2018). Practical deep stereo (pds): Toward applications-friendly deep stereo matching. Adv. Neural Inf. Process. Syst..

[53] Yu J., Fan Y., Yang J., Xu N., Wang Z., Wang X., Huang T. (2018). Wide activation for efficient and accurate image super-resolution. arXiv.

[54] Menze M., Geiger A. Object scene flow for autonomous vehicles. Proceedings of the IEEE Conference on Computer Vision and Pattern Recognition.

